# A Brominated Furanone Inhibits *Pseudomonas aeruginosa* Quorum Sensing and Type III Secretion, Attenuating Its Virulence in a Murine Cutaneous Abscess Model

**DOI:** 10.3390/biomedicines10081847

**Published:** 2022-07-31

**Authors:** Naybi Muñoz-Cázares, Israel Castillo-Juárez, Rodolfo García-Contreras, Víctor Alberto Castro-Torres, Miguel Díaz-Guerrero, José S. Rodríguez-Zavala, Héctor Quezada, Bertha González-Pedrajo, Mariano Martínez-Vázquez

**Affiliations:** 1 Laboratorio de Fitoquímica, Posgrado en Botánica, Colegio de Postgraduados, Texcoco 56230, Mexico; munoz.naybi@colpos.mx (N.M.-C.); israel.castillo@colpos.mx (I.C.-J.); 2 Departamento de Microbiología y Parasitología, Facultad de Medicina, Universidad Nacional Autónoma de México, Ciudad Universitaria, Ciudad de México 04510, Mexico; rgarc@bq.unam.mx (R.G.-C.); madiaz@ifc.unam.mx (M.D.-G.); 3 Departamento de Productos Naturales, Instituto de Química, Universidad Nacional Autónoma de México, Ciudad Universitaria, Ciudad de México 04510, Mexico; lrvictor_wilde@hotmail.com; 4 Departamento de Genética Molecular, Instituto de Fisiología Celular, Universidad Nacional Autónoma de México, Ciudad Universitaria, Ciudad de México 04510, Mexico; 5 Departamento de Bioquímica, Instituto Nacional de Cardiología Ignacio Chávez, Ciudad de México 14080, Mexico; jose.zavala@cardiologia.org.mx; 6 Laboratorio de Investigación en Inmunología y Proteómica, Hospital Infantil de México Federico Gómez, Ciudad de México 06720, Mexico; hquezadap@yahoo.com.mx

**Keywords:** *Pseudomonas aeruginosa*, type III secretion system, abscess mouse model, brominated furanone

## Abstract

Quorum sensing (QS) and type III secretion systems (T3SSs) are among the most attractive anti-virulence targets for combating multidrug-resistant pathogenic bacteria. Some halogenated furanones reduce QS-associated virulence, but their role in T3SS inhibition remains unclear. This study aimed to assess the inhibition of these two systems on *Pseudomonas aeruginosa* virulence. The halogenated furanones (*Z*)-4-bromo-5-(bromomethylene)-2(5*H*) (C-30) and 5-(dibromomethylene)-2(5*H*) (named hereafter GBr) were synthesized, and their ability to inhibit the secretion of type III exoenzymes and QS-controlled virulence factors was analyzed in *P. aeruginosa* PA14 and two clinical isolates. Furthermore, their ability to prevent bacterial establishment was determined in a murine cutaneous abscess model. The GBr furanone reduced pyocyanin production, biofilm formation, and swarming motility in the same manner or more effectively than C-30. Moreover, both furanones inhibited the secretion of ExoS, ExoT, or ExoU effectors in all tested strains. The administration of GBr (25 and 50 µM) to CD1 mice infected with the PA14 strain significantly decreased necrosis formation in the inoculation zone and the systemic spread of bacteria more efficiently than C-30 (50 µM). Molecular docking analysis suggested that the gem position of bromine in GBr increases its affinity for the active site of the QS LasR regulator. Overall, our findings showed that the GBr furanone displayed efficient multi-target properties that may favor the development of more effective anti-virulence therapies.

## 1. Introduction

The constant increase of multidrug-resistant bacteria represents one of the most significant challenges for human health [1]. To combat this global problem, the World Health Organization has issued a call to find new antibacterial drugs with novel mechanisms of action [2]. In this context, anti-virulence therapy is a plausible strategy to fight pathogenic bacteria without promoting resistance [3]. The main objective is to inhibit the production of virulence factors required to cause host damage without affecting bacterial cell viability [4,5].

Inhibition of the bacterial type III secretion system (T3SS) is an attractive approach. The T3SS is a multiprotein apparatus that facilitates the translocation of virulence effector proteins from the bacterial cytoplasm directly into a host cell [6,7]. Likewise, inhibition of the quorum sensing (QS) system has been studied among anti-virulence targets. The QS regulatory mechanisms are activated when the bacterial population reaches a threshold cell density, inducing modifications in multicellular behavior through global transcriptional changes that increase the expression of several virulence factors [8,9].

*Pseudomonas aeruginosa* QS systems have three interrelated signaling pathways, namely *las*, *rhl* and *pqs*, which regulate the production of virulence factors such as pyocyanin, rhamnolipids, alginate, alkaline protease, elastolytic activity, and also promote biofilm formation and swarming [10]. These systems are organized hierarchically. The first to be activated is the one depending on the 3-oxo-C12 homoserine lactone through binding of this signal to the LasR receptor. LasR subsequently activates the other two systems: Rhl, which depends on the C-4 signal, and PQS, which uses quinolone signals and is active in the stationary phase [11].

Inhibition of QS in *P. aeruginosa* by natural or synthetic compounds is widely documented [12,13,14]. Among the best-studied compounds are the synthetic brominated furanones C-30 and C-56, which are structurally similar to the halogenated furanones produced by the marine alga *Delisea pulchra* [15,16,17]. However, tolerance of *P. aeruginosa* clinical isolates to QS inhibitors has been reported, so the use of combined therapies exploiting QS inhibition with other targets such as the T3SS has been suggested [16,18,19].

Chemical synthesis is a practical approach to designing and developing new drugs. In the case of halogenated furanones, it has been shown that structural changes to the 2(5*H*)-furanone, such as modification of the length of the acyl chain and the pattern of halogenation in the ring, improve their capacity as anti-virulence agents [20,21,22].

In the present work, the isomeric furanones C-30 (Z-4-bromo-5-(bromomethylene)-2(5*H*)) and GBr (5-(dibromomethylene)-2-(5*H*)) were synthesized [23,24]. The GBr furanone was evaluated for its effect on QS-controlled virulence factors (pyocyanin production, biofilm formation, and swarming motility) in comparison with the previously studied furanone C-30, and on effector protein secretion by the T3SS in the *P. aeruginosa* PA14 strain and two clinical isolates (INP-42 and INP-57M). Finally, GBr was evaluated for its ability to inhibit necrosis and systemic spread of PA14 in infected mice.

## 2. Materials and Methods

### 2.1. Brominated Furanones

**Figure 1:**
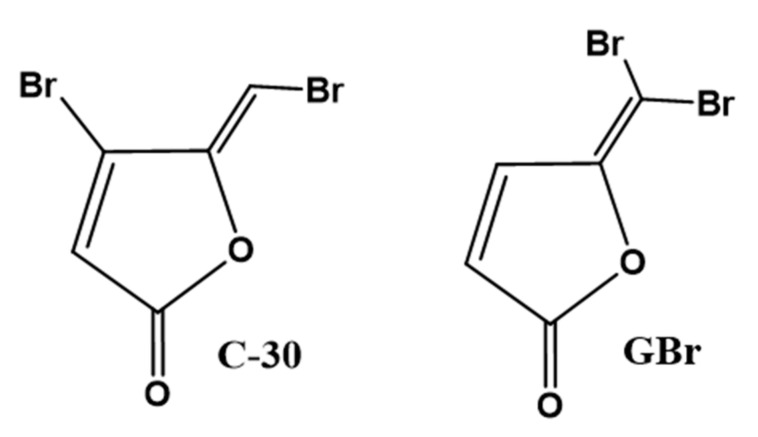
Chemical structure of C-30 and GBr furanones.

The Z-4-bromo-5-(bromomethylene)-2(5H)-furanone (C-30) and 5-(dibromomethylene)-2(5H)-furanone (GBr) (Figure 1) were synthetized as described in the Supplementary data.

### 2.2. Strains and Culture Conditions

PA14 wild-type strain originally isolated from the wound of a burn patient in the 1970s and the Δ*pscC* transposon mutant strains were obtained from Dr. Frederick Ausubel at the Harvard Medical School [25], and the Δ*lasR*/Δ*rhlR* deletion mutant was obtained from Dr. You-Hee Cho at the College of Pharmacy, CHA University, South Korea [26]. The clinical strains INP-57M and INP-42 isolated from pediatric cystic fibrosis patients were donated by Dr. Rafael Coria Jiménez from the Instituto Nacional de Pediatría, Mexico [19]. The strains were all kept in 10% glycerol at −70 °C.

The pre-cultures were grown overnight in LB broth (37 °C with 200 rpm) and the cell density was adjusted to an O.D._620nm_ of ~1.0 (UV160, Shimadzu, Japan). The furanones were added at final concentrations of 10, 50 and 100 µM. The cultures were incubated for 5 h and the production of virulence factors and growth at O.D._620nm_ were quantified. For all the assays at least three independent cultures were included. DMSO was used as a vehicle control and furanone C-30 as a positive control.

### 2.3. Virulence Factor Production

#### 2.3.1. Pyocyanin Production

Two milliliters of the cultures were centrifuged, and the supernatants were collected. Pyocyanin was extracted from the supernatant with chloroform (1:2 *v*/*v*) following re-extraction of the chloroform phase with 0.2 N HCl (3:2 *v*/*v*) [27]. The pyocyanin was determined spectrophotometrically at O.D._520nm_ (HALO MPR-96, Dynamica GmbH, London, UK) [27,28]. To calculate the inhibition percentage, the absorbance of the controls was taken as 100% of pyocyanin production.

#### 2.3.2. Biofilm Formation

Overnight cultures of the selected bacteria were diluted (1:100) in fresh LB medium to an O.D._600nm_ of 0.05, and 200 μL per well was deposited onto a 96-well plate (Corning^®^, Corning Inc., New York, NY, USA). Then, the furanones C-30 and GBr were added at final concentrations of 10, 50 and 100 µM. The plate was incubated for 24 h without shaking at 37 °C. Later, the culture was discarded, and the plate was washed with distilled water three times. The biofilm adhered to the plate was stained with 200 μL of 0.1% crystal violet for 20 min. The excess of dye was removed by rinsing with distilled water. Subsequently, 200 µL of 30% (*v/v*)-acetic acid was added and left for 15 min [29]. The adhered dye was measured at 492 nm (HALO MPR-96, Dynamica GmbH, London, UK) using acetic acid 30% as a blank. The data were normalized with respect to bacterial growth. Each assay was performed three independent times with nine replicates.

#### 2.3.3. Swarming Motility

The analysis was performed in 6-well plates (Corning^®^, Corning Inc., New York, NY, USA) with M8 minimal medium supplemented with 1 mM MgSO_4_, 0.2% glucose, 0.5% casamino acids and 0.5% agar [30]. Furanones were added to the motility agar at a final concentration of 50 µM. Aliquots (2.5 µL) were taken from overnight cultures and spotted in the center of each well, and the migration zones were measured after 24 h of incubation at 37 °C.

#### 2.3.4. Type III Secretion Assay

The type III protein secretion assay was performed as previously reported [31]. Proteins secreted to the supernatant were TCA-precipitated overnight at 4 °C and recovered by centrifugation. Type III secreted proteins were loaded onto a 15% SDS-PAGE, transferred onto a nitrocellulose membrane, and probed against anti-ExoS or anti-ExoU polyclonal antibodies. The ExoS antibodies cross-react with epitopes in the ExoT effector protein. The proteins were detected using the Immobilon Western Chemiluminescent HRP Substrate Kit (Millipore, Merck KGaA, Darmstadt, Germany) on X-ray films.

### 2.4. Docking Analysis of 3-oxo-C12-HSL and Furanones C-30 and GBr on the Binding Site of LasR

The crystal structure of LasR from *P. aeruginosa* bound to its natural autoinducer 3-oxo-C12-HSL was obtained from the protein data bank (accession no. 3IX3). The three-dimensional model of GBr used in this study was generated and optimized using Argus Lab 4.0.1 software (https://www.arguslab.com/arguslab.com/ArgusLab.html; accessed on 7 June 2022) [32] and Maestro, version 9.1 (Schrodinger, LLC, New York, NY, USA), and that of C-30 was obtained from the PubChem database (National Center for Biotechnology Information, PubChem CID = 10131246, https://pubchem.ncbi.nlm.nih.gov/compound/10131246; accessed on 19 July 2022). OCL12-HSL was derived from the LasR receptor structure using the software UCSF Chimera package 1.6 (Resource for Biocomputing, Visualization, and Informatics at the University of California, San Francisco, CA, USA; supported by NIH P41 GM103311) [33]. The LasR structure and the ligand models were prepared for docking using the software ADT 1.5.2 [34,35]. The docking analysis of LasR with the different ligands was carried out with the software Autodock 4.2.5.1 (http://autodocksuite.scripps.edu/autodock4; accessed on 7 June 2022) [36] using the genetic algorithm (GA) with the following settings: GA runs = 100, size population = 150, maximum number of evaluations = 250,000 and maximum number of generations = 27,000. Using these settings, one hundred conformations for each ligand were obtained after docking and clustered for analysis using ADT 1.5.2 software. The selected conformations corresponded to the lowest values of binding energy and inhibition constant (Ki) and were within the most represented cluster. The analysis of the resulting structures and generation of the figures were performed with PyMOL (The PyMOL Molecular Graphics System, Version 2.1.0; Schrodinger, LLC; http://github.com/schrodinger/pymol-open-source; accessed on 7 June 2022).

### 2.5. Mice Abscess Model

The mice abscess model experiment was performed as previously reported [37,38]. Briefly, six-week-old CD1 male mice were depilated using a hair remover cream (Loquay*^®^*). The mice were anesthetized with an intraperitoneal injection of pentobarbital. Prior to the injection the cultures were grown to an O.D._600nm_ of ~1.0 in LB broth and the bacterial cells were washed twice with sterile PBS. Thereafter, 60 µL of the bacterial suspension containing 1 *×* 10^8^ CFU were injected subcutaneously into the right side of the dorsum. C-30 and GBr at final concentrations of 25 and 50 µM (<2% final DMSO concentration) were diluted in the bacterial suspension and injected into the subcutaneous space with the bacteria. PBS was used as negative control. The necrotic lesion was measured every 24 h for four days.

At four days post-inoculation, the livers and the soft tissues containing the necrotic area of the mice were excised and homogenized with PBS. Serial dilutions were performed and the resulting mixtures were placed on LB plates to count colony forming units. The experiments were performed at least twice with five animals per group.

### 2.6. Statistical Analysis

The in vitro experiments were carried out at least in triplicate, and the cutaneous infection model experiments was performed at least twice. The statistical significance of the in vitro assays was evaluated using a Student’s t distribution test (* *p* < 0.05). The cutaneous infection model was analyzed by one-way ANOVA with post hoc Bonferroni corrective testing (* *p* < 0.05). All the analyses were performed with SPSS Statistics Version 25 statistical package.

## 3. Results

### 3.1. The GBr Furanone Exhibits Anti-Virulence Properties in P. aeruginosa

**Figure 2:**
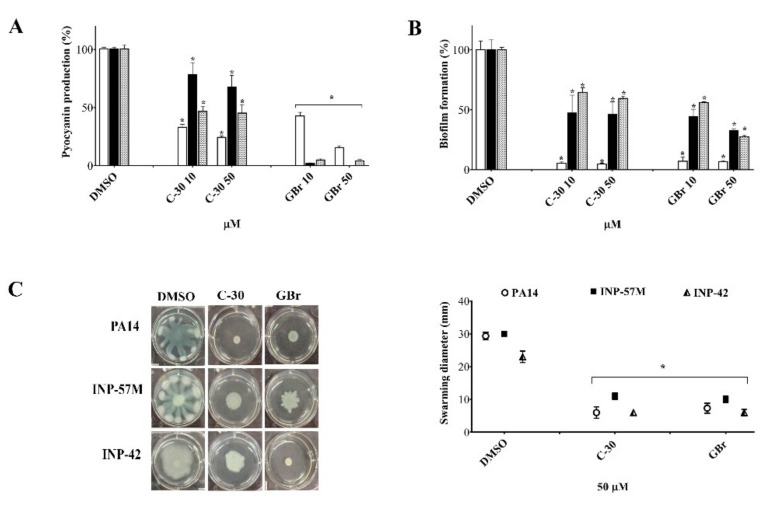
Effect of GBr and C-30 on QS regulated virulence factors in *P. aeruginosa* PA14 wild-type strain (white columns), and clinical isolates INP-57M (black columns) and INP-42 (gray columns). DMSO = dimethyl sulfoxide (vehicle). (**A**) Pyocyanin production at 10 and 50 µM furanone concentration (pyocyanin production was normalized according to the O.D._600nm_ value). (**B**) Biofilm formation at 10 and 50 µM furanone concentrations. (**C**) Swarming motility at 50 µM furanones concentration. Mean values that are significantly different from those of the control group (DMSO) are shown. Student’s *t* test for non-paired samples was used (* *p* ˂ 0.05). At least three independent experiments were carried out (* *p* ˂ 0.05).

*P. aeruginosa* produces several virulence factors positively regulated by QS. Here we analyzed the effect of the GBr furanone in comparison with C-30 (Figure 1) on pyocyanin production, biofilm formation, and swarming motility in the PA14 strain and two clinical isolates (Figure 2). Depending on the strain and the concentration used, the furanones showed differences in the inhibition of pyocyanin production (Figure 2A). Interestingly, GBr showed a more potent activity in clinical isolates than C-30, with inhibition values ranging from 20 to 100% (*p* ˂ 0.05) (Figure 2A). It should be noted that the furanones did not affect bacterial growth at 10 or 50 µM concentration, although a slight inhibitory effect was observed at 100 µM (Supplementary Figure S1).

Moreover, both furanones inhibited biofilm formation in the same way in the three tested strains. In the PA14 strain, these compounds exhibited a 90% inhibition at the two concentrations evaluated (Figure 2B). However, this activity was only reduced by 30–75% for the clinical isolates. GBr at 50 µM produced a 75% inhibition of the biofilm in the INP-42 strain (Figure 2B). In the same way, bacterial strains showed differences in the swarming patterns; nevertheless, the furanones strongly inhibited swarming motility at a 50 µM concentration (Figure 2C).

### 3.2. Furanones Inhibit the Secretion of Type III Effector Proteins

**Figure 3:**
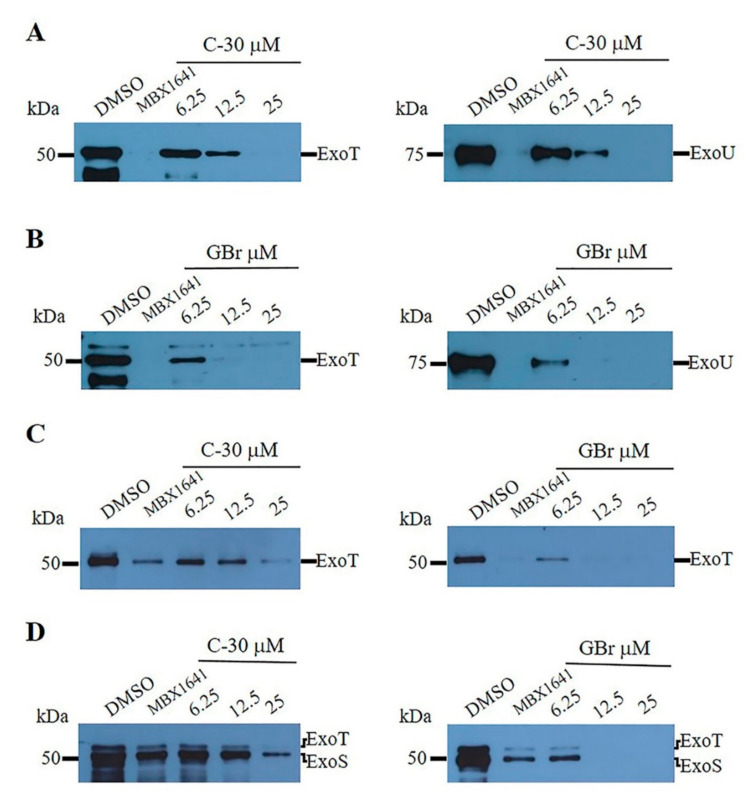
Furanones inhibit the secretion of type III effector proteins of *P. aeruginosa* strains in a dose-dependent manner. (**A**,**B**) the effect of C-30 and GBr on the secretion of ExoT and ExoU effectors in the wild-type strain PA14. (**C**,**D**) the effect of furanones on the secretion of ExoT and ExoS effectors in the clinical isolates INP-42 (**C**) and INP-57M (**D**). In the INP-42 strain, secretion of ExoS and ExoU was not detected. In the INP-57M strain, secretion of ExoU was not detected. DMSO = dimethyl sulfoxide (vehicle). MBX 1641 is a phenoxyacetamide derivative used as a positive control for T3SS inhibition (25 µM).

GBr and C-30 inhibited the secretion of type III effector proteins in the PA14 strain (ExoT and ExoU), as well as in the INP-42 (ExoT) and INP-57M (ExoT and ExoS) clinical strains (Figure 3). The furanones did not significantly affect bacterial growth under the secretion assay conditions (Supplementary Figure S2). Our results indicate that GBr and C-30 at a 25 µM concentration blocked the secretion of these toxins in all three strains. In all cases, the GBr furanone showed a stronger effect with complete inhibition at 12.5 µM concentration (Figure 3A–D). Remarkably, GBr at 25 µM also showed better efficacy than the commercial inhibitor MBX1641 for the clinical strain INP-57M (Figure 3D).

### 3.3. Effect of GBr on a P. aeruginosa-Mouse Infection Model

**Figure 4:**
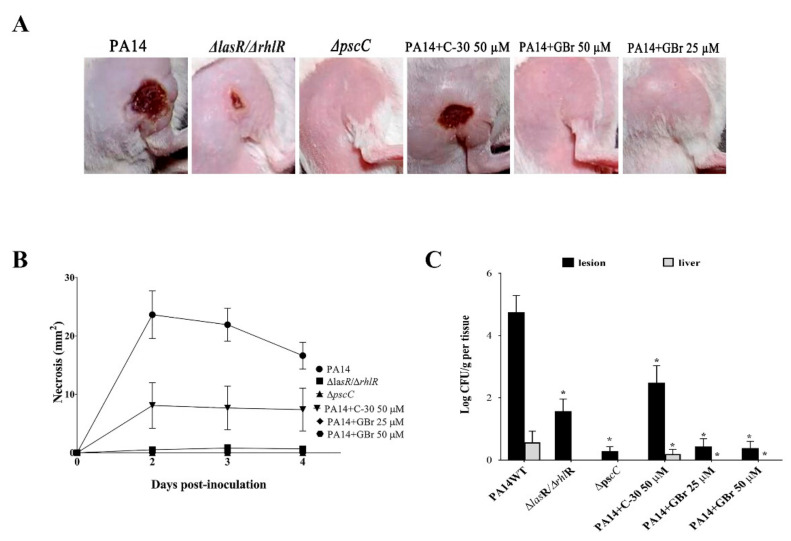
Effect of halogenated furanones on the establishment and induction of necrosis of *P. aeruginosa* PA14. Compounds were incubated with 1 × 10^8^ CFU for 20 min and injected subcutaneously into mice. (**A**) Representative images of the inoculation areas at 48 h, the time in which the greatest formation of the necrotic area was recorded. (**B**) Induction of necrotic tissue over time. (**C**) Bacterial load in the lesion and liver on the fourth-day post-inoculation. (* Significant differences one-way ANOVA with post hoc Bonferroni corrective testing, *p* < 0.05). Three independent experiments were carried out with groups of five animals. Strains Δ*lasR/*Δ*rhlR* and Δ*pscC* were used as negative controls. CFU: colony-forming units. The error bars represent the standard error.

Previous reports showed that subcutaneous inoculation of mice with *P. aeruginosa* induces abscess formation, necrosis, and death [37,38]. Using this model, we evaluated the protective effect of GBr in comparison with C-30. The PA14 mutant strains Δ*lasR/*Δ*rhlR* (lacking the QS regulators LasR and RhlR) and Δ*pscC* (the T3SS is not assembled) were used as negative controls (Figure 4).

The subcutaneous injection of the PA14 wild-type strain in CD1 animals elicited the formation of a necrotic area at 48 h (Figure 4A) and caused the death of 26% of mice in the first four days. As expected, the Δ*lasR/*Δ*rhlR* and Δ*pscC* mutants did not form lesions or these were much smaller than those generated by the PA14 strain (Figure 4A,B). A similar effect was observed with inoculation of PA14 plus GBr at 25 and 50 µM, where the ability of the bacteria to induce necrotic areas was abolished; whereas with C-30 at 50 µM, the size of the lesions was only reduced compared with the control without treatment (Figure 4A,B).

Moreover, GBr significantly reduced the presence of bacteria in the inoculation zone, prevented systemic dissemination, and maintained 100% survival of the animals. Interestingly, the reduction of bacterial load in the lesions was significantly larger in the presence of GBr 25 and 50 µM, compared with C-30 at 50 µM (Figure 4C). The viability of the inoculum in the presence of the furanones was corroborated (Supplementary Figure S3). The remarkable efficiency of GBr as an anti-virulence drug was also evident in the reduction in the bacterial burden in the lesions after 96 h and in the absence of systemic spread when used at 25 or 50 µM (Figure 4C). In the presence of GBr, the number of bacterial cells in the abscess was similar to that observed in the Δ*pscC* mutant, and no bacteria were detected in the liver, indicating that GBr contributed to containing the primary infection. In contrast, C-30 at 50 µM failed to prevent the systemic spread and only reduced the bacterial burden to about half of that observed in the controls (Figure 4C).

### 3.4. Molecular Docking Analysis

**Figure 5:**
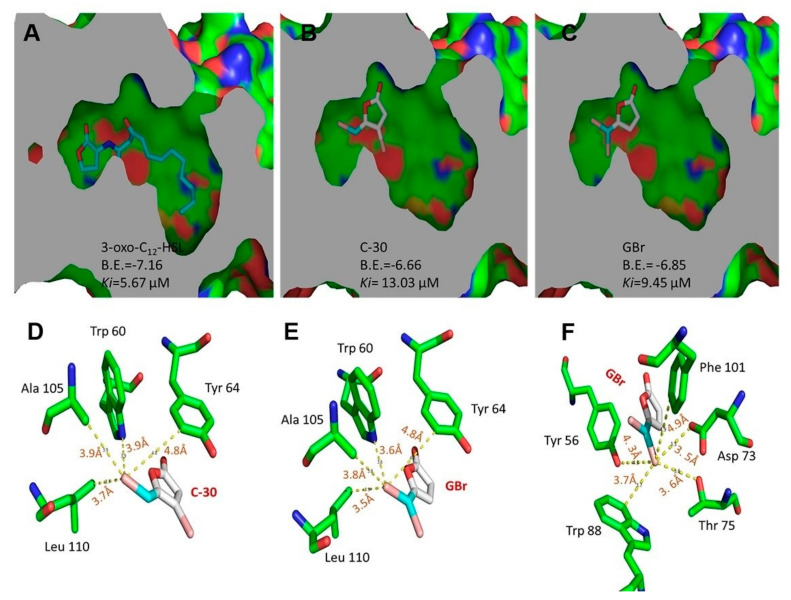
Slice of the binding site of the LasR receptor protein showing interactions revealed by docking with the autoinducer 3-oxo-C12 homoserine lactone (**A**), furanone C-30 (**B**) and GBr (**C**). Interactions of Br atom at C5 of C-30, with amino acids at less than 5 Å in the binding site of LasR (**D**). Interactions of Br “a” (**E**) and “b” (**F**) at C5 of GBr with amino acids in the binding site of LasR.

C-30 is a synthetic derivative whose structure resembles that of furanones isolated from *D. pulchra*, the first described molecules with QS inhibition activity [15,16]. The proposed mechanism of action of C-30 involves antagonism to the LasR transcriptional regulator [15,39]. GBr is a structural isomer of C-30 but varies in the gem position of the bromines (Figure 1). Molecular docking analysis showed that the binding site of the LasR receptor has a higher theoretical affinity for GBr (B.E. = −6.85, *Ki* = 9.45 µM) compared with C-30 (B.E. = −6.66, *Ki* = 13.03 µM), although none was greater than the theoretical affinity determined for the natural autoinducer, the 3-oxo-C12-HSL (B.E. = −7.16, Ki = 5.67 µM) (Figure 5A–C). Similarly, the change of position of the Br to C5 in GBr allowed the establishment of interactions with other amino acids within the active site, such as Tyr 56, Asp 73, Trp 88, Phe 101, and Thr 75 (Figure 5F).

## 4. Discussion

Furanones consist of a five-membered heteroaromatic ring (furan) containing an oxygen atom and are produced by many living organisms such as plants, fungi, and algae [40]. Specifically, halogenated alkyl-furanones (fimbrolides) produced by the macroalga *D. pulchra* were the first reported molecules with quorum quenching (QQ) properties [24,41]. However, it should be noted that prior to this discovery, synthesis reactions of various halogenated furanones, such as C-30 and GBr, were already described [23].

**Figure 6:**
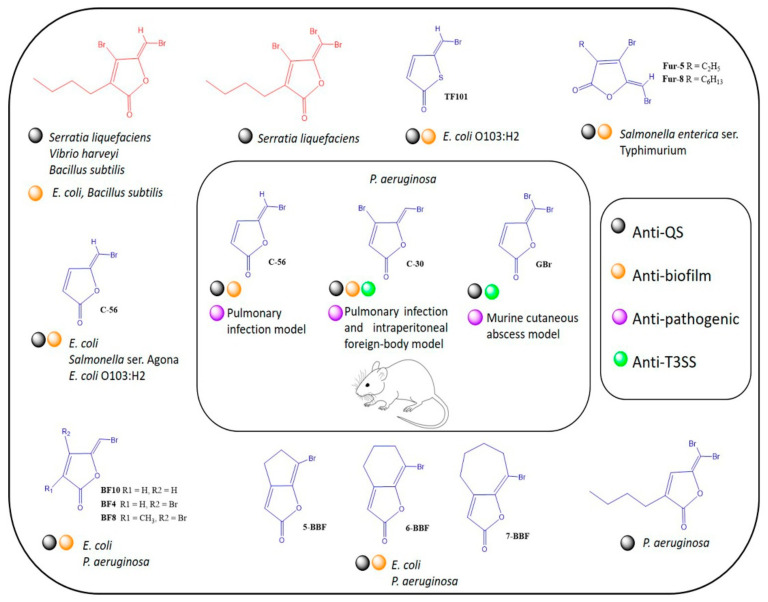
Overview of the anti-virulence properties of halogenated furanones. In the 1990s, the anti-QS properties of halogenated furanones isolated from the macroalgae *Delisea pulchra* (4-Bromo-5-(bromomethylene-3-butyl-2(5*H*)-furanone and 4-Bromo-3-butyl-5-(dibromomethylene-2(5*H*)-furanone) began to be described (red molecules) [41,53,54,55]. Subsequently, chemical synthesis and modifications have generated many synthetic furanones (blue molecules) that have enhanced anti-QS and anti-biofilm capacity, mainly in in vitro assays [39,56,57,58,59,60,61]. However, few preclinical trials have been performed with murine models and have not shown to eliminate the infection [15,17,62]. One possible explanation is that bacteria such as *P. aeruginosa* contain several QS systems and complex regulatory mechanisms that allow them to compensate the inhibition of one QS system and remain virulent [40]. The problem is more remarkable as the regulation of the T3SS in *P. aeruginosa* appears to be QS-independent as it remains active in QS mutants [63]. This phenomenon makes the development of effective anti-virulence therapies a complicated task. In this investigation, we report the ability of furanone GBr to inhibit the T3SS of *P. aeruginosa* and its anti-pathogenic capacity in a murine infection model. The ability of GBr to inhibit QS and the T3SS opens a new window to study the multi-target properties of halogenated furanones.

In recent years, the synthesis of furanone derivatives and the exploration of their anti-virulence properties have been of great interest [22,42]. C-30 furanone has become the reference anti-QS molecule, whose mechanism of action involves the competition of acyl-homoserine-lactones (HSL) signals with their receptors [31,38,39,43]. Similarly, it has been shown to reduce the severity of lung damage caused by *P. aeruginosa* in a mouse model [17]. However, in the case of GBr, its anti-virulence capacity has been less explored, and even null or low activity has been reported in some bacterial genera such as *Salmonella* and *Vibrio harveyi* [22]. Previous literature concerning halogenated furanone-based QS inhibition is presented in Figure 6.

The QS and T3S systems are involved in the induction of pathogenicity in bacteria, so their inhibition is essential for developing more robust anti-virulence strategies [18,44]. Although QS systems are proposed as the main determinants of virulence in bacteria, their role in regulating bacterial secretion systems is not fully understood [45].

The T3SS is a complex syringe-shaped structure of more than 20 proteins located in the cytoplasm, membranes, and external environment of some pathogenic bacteria. It injects a series of proteins called effectors into the cytosol of the infected cell, severely affecting its homeostasis [6,7,44]. Although only four major effectors have been identified in *P. aeruginosa* (ExoS, ExoT, ExoU, and ExoY), they rarely coexist in a single strain, and some may have either the *exoS* or the *exoU* gene. The expression of these effectors has been correlated with clinical infections, and ExoS has been reported to induce apoptotic cell death, while ExoU induces cell lysis [46].

In this study, we synthesized furanone C-30 and the GBr isomer and compared their ability to inhibit the major virulence factors controlled by QS and the secretion of effector proteins by the T3SS. Our results corroborated that synthetic furanones inhibit the production of QS-regulated virulence factors. Comparing our results with previous work, the PA14, INP-57M and INP-42 strains produced similar levels of pyocyanin; however, its inhibition by C-30 is stronger in this work [19]. This difference may be related to slight modifications in the administration of the compound, since in the previous work, C-30 was dissolved in ethanol instead of DMSO, and was added until the cultures reached an O.D._600nm_ of 1.0. Moreover, our results show that the furanones also inhibit type III effector protein secretion in strain PA14 and the clinical isolates INP-57M and INP-42, and remarkably, GBr was more effective than C-30 in the clinical strains.

In the last decade, many QQ molecules and other anti-virulence compounds have been described, but confirmation of their efficacy in mammalian infection models remains scarce [3]. In this regard, in some mouse models, such as lung infection and thermally induced injury, some QQ molecules have been shown to reduce damage, systemic spread, and death [17,47,48]. Recently, the abscess-necrosis model has been used, showing good reproducibility [37,38]. In this model, we show that GBr 25 µM reduced pathogenicity in vivo and has a better effect than C-30 (50 µM) in reducing necrosis, the establishment of infection, and systemic spread.

The bactericidal and toxic properties of halogenated furanones have been documented and represent one of the main obstacles to their use in designing anti-virulence therapies [42,49]. In the case of secretion assays, a slight growth retardation at a 25 µM concentration was observed in all strains (Supplementary Figure S2). However, it should be noted that in the in vivo model, pretreatment with 25 and 50 μM GBr for 20 min before subcutaneous administration did not affect the viability of bacteria (Supplementary Figure S3).

The QQ mechanism of C-30 furanone is known to involve interference with autoinducer LasR recognition [15,21]. However, the QQ and anti-virulence properties of GBr have been poorly investigated [22,24]. The GBr derivative is a structural isomer of C-30, so we consider it likely that it acts with a similar QQ mechanism. In this study, we showed that changing the position of a Br-C4 atom to the gem position favors the anti-virulence activity and reduces its bactericidal properties (Supplementary Figure S1). This may be due to a higher affinity of GBr for the binding site of the LasR protein, as suggested by molecular docking analysis. We suggest that an additional Br at the 5-methylene residue in GBr, compared with C-30, promotes the formation of new interactions with the LasR binding site, specifically at Tyr 56, Asp 73, Trp 88, Phe 101, and Thr 75. Similarly, this could partly explain why the theoretical affinity of LasR for GBr is higher than that for C-30. Therefore, we suggest that the new interactions formed with Br at position 5 are more critical for binding to the receptor than those lost by the absence of Br at position 4. This is consistent with previous studies with 3-alkyl-5-methylene 2(5*H*)-furanones that have reported that the acyl chain length and bromination pattern of the furan ring are critical for reducing biofilm formation in *Salmonella* and for QQ properties in *V. harveyi* [22,42].

Furthermore, although it is known that some furanones act as antagonistic molecules, interfering with the recognition of the autoinducer by the transcriptional regulator [13,50], other QQ or anti-virulence mechanisms cannot be ruled out. In this regard, it has been reported that some furanones participate in the degradation or induction of conformational changes in the LasR protein, favoring its recognition by cellular proteases [43]. Similarly, in some gene expression studies using microarrays, it was observed that C-30 affected the expression of 93 genes out of 5570 analyzed in the PAO1 strain. Of these, 83 genes were repressed in the presence of the furanone, some of them related to QS-regulated virulence factors (*lasB*, *lasA*, *rhlAB*, *phzA-G*, *hcnABC*, and *chiC*), but around 43% were of unknown function [15].

As mentioned above, the complex interplay between QS and secretion systems is not yet fully understood. In this context, our results are relevant since some studies indicate that T3S and QS systems are regulated independently and that the T3SS remains active even when QS is interrupted [45,51]. In contrast, other reports indicate that QS exerts a negative regulation on the expression of the T3SS [52]. In both cases, the efficacy of anti-virulence therapy with QQ molecules would be compromised since the bacterium will remain virulent with an active T3SS. Therefore, the development of anti-virulence strategies with multi-target molecules would be advantageous.

## 5. Conclusions

In conclusion, the multi-target properties of halogenated furanones have been little explored, but the results of this research provide evidence that they have this ability. Specifically, although the GBr furanone had gone unnoticed among many other synthetic derivatives, it is a potential candidate for designing and developing more effective anti-virulence therapies against *P. aeruginosa*.

## Data Availability

Data is contained within the article.

## Supplementary Materials

The following supporting information can be downloaded at: https://www.mdpi.com/article/10.3390/biomedicines10081847/s1. Figure S1: Effect of furanones on the growth of *P. aeruginosa* strains under conditions of the assays used for evaluation of QS-regulated virulence factors; (A) PA14, (B) INP-57M and (C) INP-42. Figure S2: Effect of furanones on the growth of *P. aeruginosa* strains under the conditions used for the type III secretion assay; (A) PA14, (B) INP-57M and (C) INP-42; MBX1641 is a phenoxyacetamide derivative used as a positive control for T3SS inhibition (25 μM). Figure S3: Effect of furanones on the viability of the bacterial inoculum used in infection assays in mice. The cultures were adjusted to the different concentrations of furanones and incubated for 20 min. Subsequently, 60 μL was used for animal inoculation.
