# Supplementary material for: A Brominated Furanone Inhibits Pseudomonas aeruginosa Quorum Sensing and Type III Secretion, Attenuating Its Virulence in a Murine Cutaneous Abscess Model
Source: Biomedicines. 2022 Jul 31;10(8):1847. doi: 10.3390/biomedicines10081847 (PMC9404868; doi:10.3390/biomedicines10081847)
Supplement: Supplementary file 1 [file biomedicines-10-01847-s001.zip › biomedicines-1786273-supplementary.pdf]

Supplementary material

A Brominated Furanone Inhibits *Pseudomonas aeruginosa* Quorum Sensing and Type III Secretion Attenuating Its Virulence in a Murine Cutaneous Abscess Model

Naybi Muñoz-Cázares, Israel Castillo-Juárez, Rodolfo García-Contreras, Víctor Alberto Castro-Torres, Miguel Díaz-Guerrero, José S Rodríguez-Zavala, Héctor Quezada, Bertha González-Pedrajo* and Mariano Martínez-Vázquez*.

Brominated furanones

Synthesis of *Z*-4-bromo-5-(bromomethylene)-2(5*H*)-furanone (C-30) (Figure 1) was achieved starting from the precursor 3,5-dibromolevulinic acid which was prepared as follows: A solution of Br_2 (3.56 g, 0.022 mol), in CH_2Cl_2 (10 mL) was added drop wise to a solution of levulinic acid (1.23 g, 0.010 mol) and 20 drops of HBr (45% in H_2O) in CH_2Cl_2 (100 mL) at 0° C. The solution was stirred vigorously at room temperature for 2 h. Once the reaction was ended, the mixture was washed with water (50 mL) and with a saturated solution of $Na_2S_2O_3$ (50 mL). The aqueous phase was extracted with CH_2Cl_2 (4 x 20 mL). The organic phase was dried with anhydrous Na_2SO_4. Hexane was added under vigorous stirring until a precipitate was observed, allowed to stand on ice for 15 min and filtered.

The 1.62 g of 3,5-dibromolevulinic acid obtained in the previous step was dissolved in 15 ml of concentrated H_2SO_4 and the reaction was heated at 90-100° C for 20 minutes. The resulting dark oil was poured onto ice, and it was extracted with CH_2Cl_2 (4 x 20 mL), washed with $NaHCO_3$ (50 mL) and H_2O (50 mL). The organic phase was dried with anhydrous Na_2SO_4 and concentrated under reduced pressure to obtain a yellow oil which was subjected to a chromatographic column (Hex/AcOEt 9:1) to obtain the pure white crystalline solid (0.6 g, 40%). Mp 104-105° C. MS, *m/z* (DART+): 254.84779 Anal. Calcd for $^{12}C_5{}^{1}H_3{}^{79}Br_1{}^{81}Br_1{}^{16}O_2$. RMN^{1}H (300 MHz, $CDCl_3$) d 6.50 (s, 1H), 6.41 (s, 1H); RMN^{13}C (75 MHz, $CDCl_3$) d 165.5, 151.2, 135.3, 121.1, 93.8.

Synthesis of 5-(dibromomethylene)-2(5*H*)-furanone (GBr) (Figure 1): 3,5-dibromolevulinic acid (4.77 g, 17.4 mmol) was dissolved in concentrated H_2SO_4 (10 mL) with gentle stirring and at room temperature. The mixture was then placed in an oil bath and stirred vigorously at a temperature of 50-60° C for 10 min and it was left stand until it reached room temperature. The dark oily product was poured onto ice and extracted with CH_2Cl_2 (3 x 20 mL). The organic phases were collected, washed with distilled water, and dried with anhydrous Na_2SO_4. The solvent was removed under reduced pressure and the residue obtained was subjected to a chromatographic column with hexane/AcOEt (9:1) to give the pure compound as orange crystals. Mp 124–126° C. MS, *m/z* (DART+): 254.84750. Anal. Calcd for $^{12}C_5{}^{1}H_3{}^{79}Br_1{}^{81}Br_1{}^{16}O_2$. RMN^{1}H (300 MHz, $CDCl_3$) d 7.67 (d, *J* = 5.5 Hz, 1H), 6.41 (d, *J* = 5.6 Hz, 1H); RMN^{13}C (75 MHz, $CDCl_3$) d 167.90, 150.91, 140.84, 122.58, 81.98.

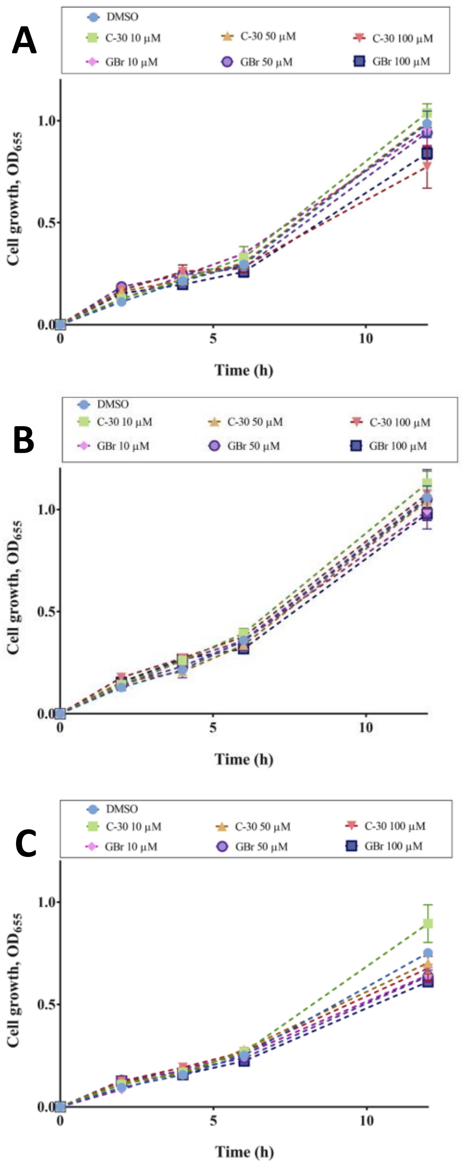

Figure S1. Effect of furanones on the growth of *P. aeruginosa* strains, under conditions used for evaluation of QS-regulated virulence factors. (**A**) PA14, (**B**) INP-57M and (**C**) INP-42.

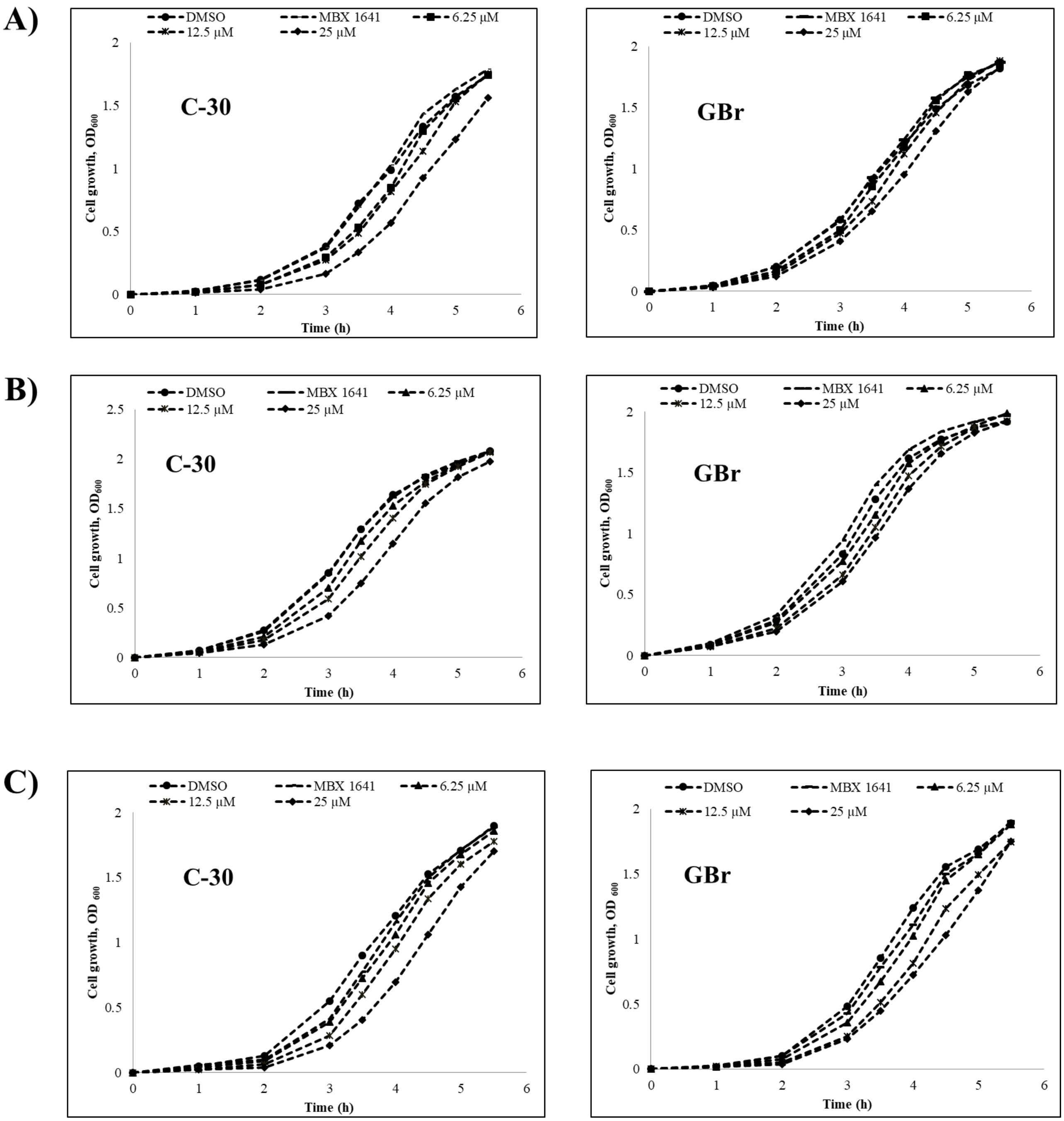

Figure S2. Effect of furanones on the growth of *P. aeruginosa* strains under conditions used for the secretion assays. (**A**) PA14, (**B**) INP-57M and **(C)** INP-42. MBX1641 is a phenoxyacetamide derivative used as a positive control for T3SS inhibition (25 µM).

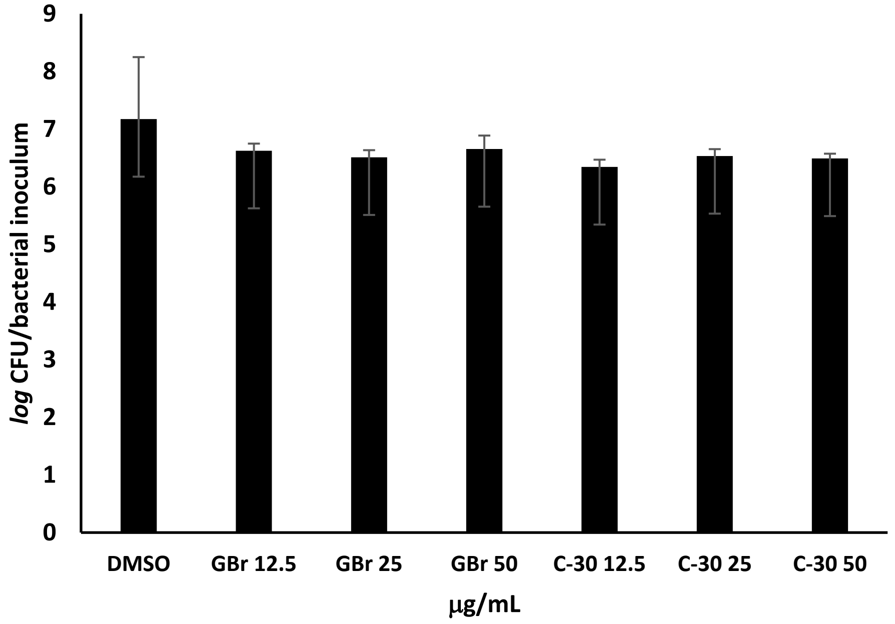

Figure S3. Effect of furanones on the viability of the bacterial inoculum used in infection assays in mice. The cultures were adjusted to the different concentrations of furanones and incubated for 20 min. Subsequently 60 µl was used for animal inoculation.
